# Diffuse myocardial fibrosis in pediatric hypertrophic cardiomyopathy

**DOI:** 10.1186/1532-429X-15-S1-O72

**Published:** 2013-01-30

**Authors:** Tarique Hussain, Andrea Dragulescu, Lee Benson, Derek Wong, Mark Friedberg, Luc Mertens, Shi-Joon Yoo, Lars Grosse-Wortmann

**Affiliations:** 1 Cardiology, Birmingham Children's Hospital, Birmingham, UK; 2 Cardiology, Hospital for Sick Children, Toronto, ON, Canada; 3 Children's Hospital of Eastern Ontario, Ottawa, ON, Canada

## Background

Fibrosis is a common end-point in pathological processes. It is unclear, however, if diffuse fibrosis occurs early in the pathogenesis of hypertrophic cardiomyopathy (HCM). The purpose of this study was to evaluate the presence of diffuse myocardial fibrosis in children and adolescents with HCM using quantification of T1 changes late after gadolinium administration and to assess for associations between markers of fibrosis and standard clinical parameters of disease.

## Methods

**Figure 1:**
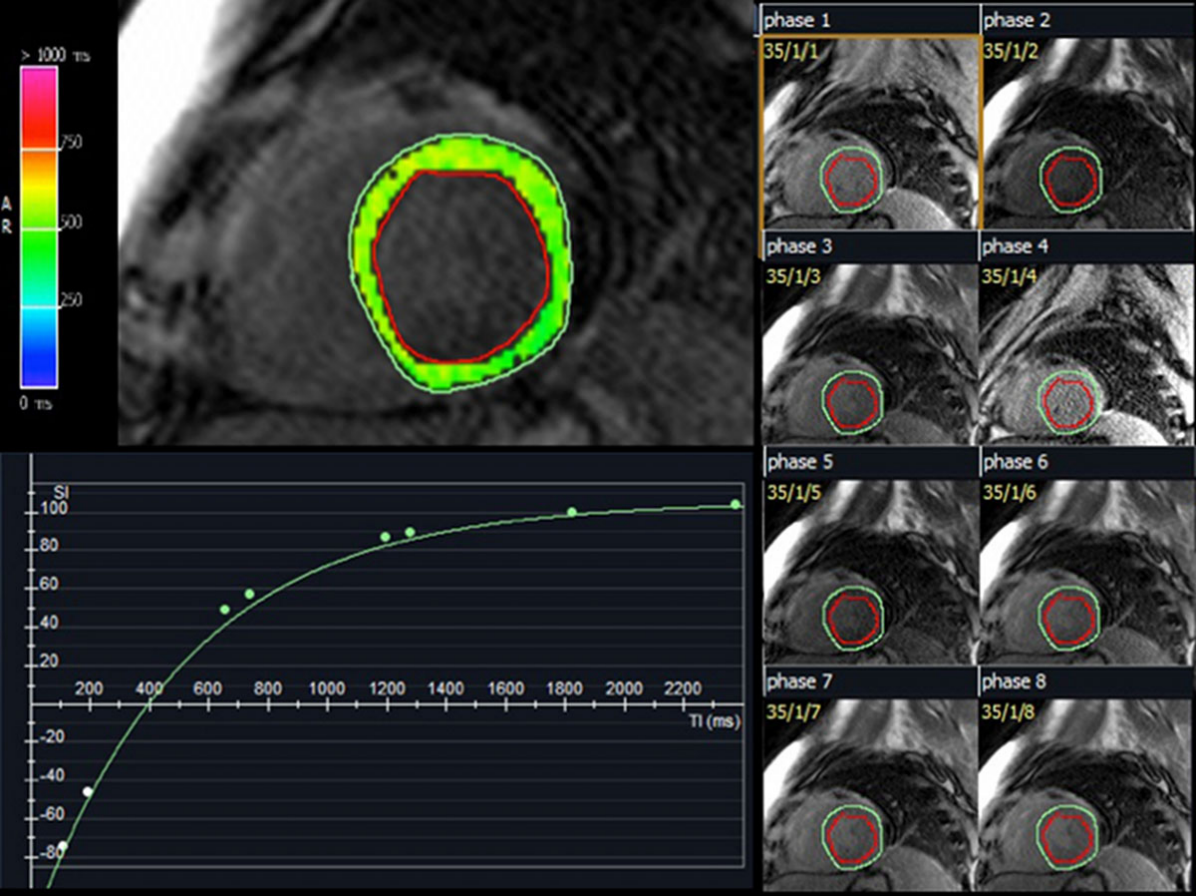
A single mid-ventricular slice is imaged over multiple inversion times. A curve-fitting technique is used to create a T1 map of the myocardium. Individual regions of interest in the septum, blood pool and lateral wall are subsequently drawn and recorded.

Patients with confirmed HCM and healthy controls participated in this study. T1 measurements were made using standard multi-breath-hold spoiled gradient echo phase-sensitive inversion-recovery MRI. They were performed before and 15 minutes after 0.2 mmol/kg gadopentetate dimeglumine in a single mid-ventricular slice using increasing inversion times per breath-hold (150, 400, 800 and 3200 ms). For analysis, regions of interest were drawn in the septum, blood pool and left ventricular (LV) lateral wall, avoiding myocardium showing overt late enhancement (LGE). A curve-fitting technique was used to derive the T1 time constant (Figure 1). The tissue-blood partition coefficient (PCf) was calculated as a function of the ratio of T1 change of myocardium compared to blood [1]: higher PCf values indicate greater fibrosis. Clinical data included LV mass, ejection fraction, presence of LGE, symptoms, serum brain-natriuretic-peptide (BNP), mitral valve inflow velocities and presence of outflow tract obstruction at rest.

## Results

12 controls (mean age 12.8yrs; 7 male) and 28 patients (mean age 12.8yrs; 21 male) participated. All patients had a clinical diagnosis of HCM. Among these, 20 children had HCM-specific mutations. Clinical parameters are given in table 1. PCf for both septal (0.27±0.17 vs. 0.13±0.09 ml/g; p=0.03) and lateral walls (0.22±0.09 vs. 0.07±0.10; p<0.001) were increased in patients compared to controls. PCf did not correlate with age, either in patients or normal individuals.

**Table 1:** Parameters in Controls vs. HCM Patients

	Controls (mean ± s.d.)	HCM Patients	p-value (*<0.05)
Number	12	28	
No. male	7	21	
Age (yrs.)	12.8 ± 2.2	12.8 ± 2.6	0.95
Septum partition coefficient (ml/g)	0.13 ± 0.09	0.27 ± 0.17	0.03*
Lateral wall partition coefficient (ml/g)	0.07 ± 0.10	0.22 ± 0.09	<0.001*
BNP (pg/ml)	10.1 ± 6.6	283 ± 429	0.009*
Number with LGE	0	8	
Ejection fraction (%)	57 ± 3	68 ± 10	<0.001*
Indexed myocardial mass (g/m^2^)	54 ± 7	103 ± 40	<0.001*

Note that the partition coefficients are significantly raised in HCM patients compared with controls. (BNP = Brain Natriuretic Peptide; LGE = Late Gadolinium Enhancement)

Eight patients had overt areas of LGE. These patients did not show increased coefficients compared to those without LGE (0.27±0.15 vs. 0.27±0.19 and 0.22±0.09 vs. 0.22±0.09; p=0.95 and 0.98 respectively). However, patients that were symptomatic (dyspnoea, arrhythmia or chest pain) had higher lateral wall coefficients than asymptomatic HCM patients (0.27±0.08 vs. 0.17±0.08; p=0.006). Similarly, patients with raised BNP (>100 pg/ml) had raised lateral wall coefficients (0.27±0.07 vs. 0.20±0.07; p=0.03). Other clinical parameters did not show any discrimination with respect to the lateral coefficient.

## Conclusions

Diffuse fibrosis, demonstrated by the partition coefficient technique, is demonstrable in children and adolescents with HCM. Markers of fibrosis show an association with symptoms and raised serum BNP. Further study of the prognostic implication of this technique is warranted.

## Funding

No specific funding was given for this research.
